# Correlation Analysis between Strength and Defect of Nano-Cementitious Composites using Ultrasonic Pulse Velocity

**DOI:** 10.3390/nano13071183

**Published:** 2023-03-27

**Authors:** Yangsub Shin, Sanghyeon Cho, Hyojeong Yun, Wonseok Chung

**Affiliations:** Department of Civil Engineering, Kyung Hee Univeristy, 1732 Deokyoung-Daero, Giheung-gu, Yongin-si 17104, Republic of Korea

**Keywords:** multi-walled carbon nanotube, non-destructive analysis, defect, ultrasonic pulse velocity, compressive strength

## Abstract

Recently, researchers are conducting studies to improve the mechanical and chemical properties of cementitious composites mixed with nanomaterials. Defects may occur inside nano-cementitious composites due to nanomaterial agglomeration in the manufacturing process. These defects can degrade the mechanical performance of the nano-cementitious composite. This study performs ultrasonic non-destructive and compressive strength tests according to the size of defects in nano-cementitious composites. Multi-walled carbon nanotubes (MWCNTs) were used for the nanomaterial, and internal defects of various sizes were considered in the center of the specimens. Ultrasonic pulse velocity was measured according to the defect size until 30 curing days, after which the compressive strength was measured. The ultrasonic pulse velocity of the nano-cementitious composites decreased by up to 9.6% in relation to that of the specimens without defects as the defect size increased, and the compressive strength decreased by up to 35.7%. This study’s findings revealed a correlation between ultrasonic pulse velocity and compressive strength according to defect size. Future ultrasonic non-destructive tests will allow for the prediction of mechanical performance and the detection of defects within nano-cementitious composites.

## 1. Introduction

Studies have recently been conducted to mix nanomaterials into construction materials and improve their versatility [1,2,3,4,5,6,7,8,9,10,11,12,13,14,15,16,17]. Particularly, researchers worldwide are performing studies on the electrical properties and heating performance of cementitious composites using nanomaterials. Yoo et al. [3] investigated the effect of nanomaterials on the piezoresistive sensing capacity of nano-cementitious composites. The sensing capacity was related to the electrical properties of nanomaterials. Yoo et al. [4] evaluated the effect of the carbon-based nanomaterials type on electrical properties of nano-cementitious composites. According to previous studies, multi-walled carbon nanotubes (MWCNTs) was considered to be the most effective to improve the self-sensing capacity of the cementitious composites. MWCNTs was also used for research on heating road pavement to prevent black ice. Defects may occur inside these nano-cementitious composites due to faults in the fabrication process and poor dispersion of the nanomaterials. Cheng et al. [5] found that inappropriate vibrations and excessive dry mixing cause defects in nano-concrete structures. Researchers characterized the dispersion MWCNT solution using atomic-force microscopy, scanning electronic microscope, transmission electron microscopy. The dispersibility of the MWCNT solution needed to be secured because defects could occur when the MWCNT solution was mixed unevenly into concrete. Yu and Lau [6] summarized the experimental works on concrete mixed with MWCNTs covering dispersion, mechanical performance, and microstructures. Collins et al. [7] studied the dispersion and rheology of nano-cementitious composites according to dispersants and surfactants. Chen et al. [8] emphasized the importance of the dispersion of MWCNTs which improved the bridging efficiency and mechanical performance of the nano-cementitious composite. Dispersion of MWCNTs must be secured to maximize the mechanical and electrical properties when mixing MWCNTs into a cement-based structure. The strong van der Walls force generated between MWCNT particles interferes with the maintenance of dispersibility, leading to agglomeration. Kang et al. [9] applied acid treatment to maximize the strength enhancement of nano-cementitious composites by improving the dispersion of MWCNTs and adhesion onto the cement. Elkashef and Abou-Zeid [10] studied the effect of the surfactant-to-MWCNT ratio on dispersion efficiency by comparing mechanical performances. MWCNTs had a high probability of clumping due to their high van der Waals interactions and also exhibited extremely low dispersibility in water due to their strong hydrophobic nature [11]. Chen et al. [12] studied the characteristics of MWNCT including the stability of the dispersion and morphology of the agglomerates. Research on analyzing and detecting defects is necessary because defects caused by these reasons degrade the structure’s stability and versatility.

To assess internal conditions in the concrete structure, a variety of non-destructive tests have been carried out. It is possible to detect defects by comparing the change in temperature with thermal images in cementitious composites mixed with carbon-based nanomaterials induced during heat generation performance measurement experiments. Moreover, it is possible to detect defects by measuring electrical resistance when carbon-based nanomaterials were mixed in a cementitious composite, mortar, and concrete [13,14,15]. Lee et al. [16] analyzed the relationship between the filling rate and pore of the cement grout mixed with MWCNT. The specimen was measured for electrical resistance according to the MWCNT concentration and grout filling rate. As a result of the experiment, the ordinary Portland cement (OPC) specimen did not show a clear difference in electrical resistance according to the filling rate. The specimen containing 0.1 wt% of MWCNT showed a clear difference in electrical resistance according to the filling rate, but the electrical resistance was measured to be similar to that of the OPC specimen. In the specimen containing 1.0 wt% of MWCNT, the electrical resistance increased as the filling rate decreased. The electrical resistance of the specimen containing 1.0 wt% MWCNT was measured 1000 times smaller than the 0.1 wt% specimens. There was little electrical conductivity in the OPC specimen and specimens mixed with 0.1 wt% MWCNT because of its high electrical resistance, so pore detection was difficult. Most studies on analyzing defects caused by mixing in nanomaterials depended on heat generation and electrical performance, and there is insufficient research on non-destructive tests to verify defect formation.

Representative concrete non-destructive inspection methods include the rebound hardness method, the radiographic test, and the ultrasonic pulse velocity (UPV) method. The rebound hardness method is a concrete strength estimation technique that calculates the rebound coefficient by striking the concrete surface. The radiographic test uses x-rays, gamma rays, and other radiation to look for internal defects or rebars. The UPV method is the most widely used non-destructive test that can analyze the conditions of concrete using ultrasonic. Related regulations are specified in the “Standard Test Method for Pulse Velocity Through Concrete (ASTM C 597)” [17]. Related studies are actively underway owing to the UPV method’s simplicity [18]. Chang et al. [19] analyzed lightweight concrete through several non-destructive tests using ultrasonic and derived engineering properties of concrete, such as strength and moduli of elasticity. Hadianfard et al. [20] measured ultrasonic pulse velocity and conducted non-destructive tests on concrete and were able to predict the compressive strength via ultrasonic pulse velocity by constructing an algorithm. Qurashi et al. [21] performed compressive strength tests, ultrasonic non-destructive tests, and the rebound hardness method to analyze the relationship between the destructive and non-destructive tests according to the curing days of concrete. According to the test results, it was possible to predict the mechanical performance of concrete within an error range of 8% by using the non-destructive test results on all curing days. The amplitude was compared to detect damage by analyzing the signal attenuation in damaged concrete with ultrasonic [22]. Researchers predicted damage by comparing reflected signals, such as front wall echo, defect echo, and back wall echo in concrete pipes through ultrasonic non-destructive tests [23]. In a mortar, the internal porosity and compressive strength were estimated by measuring ultrasonic pulse velocity, even when mixed with lightweight materials [24]. In thermally damaged concrete, the features of internal damage and pores were distinguished by comparing the ultrasonic signals with scanning electron microscopy (SEM) images [25]. Saint-Pierre et al. [26] collected the core of an actual dam to analyze the damage in the spillway concrete pier. The ultrasonic pulse velocity measured in the core was utilized as a factor for judging concrete quality designation (CQD). In reinforced concrete, researchers detected cracks caused by rebar corrosion by measuring the ultrasonic signal and comparing the amplitude and frequency [27]. A study also detected and estimated the dynamic moduli of elasticity and detected delamination in reinforced concrete using ultrasonic pulse velocity [28]. Similarly, ultrasonic was used to detect carbon fiber reinforced polymer (CFRP) debonding in concrete reinforced with CFRP as well [29]. Rathod et al. [18] found that ultrasonic pulse velocity was more effective than other non-destructive tests for distinguishing surface damage in reinforced concrete slabs. In addition to the UPV method, tests using ultrasonic such as the phased array technique and total focusing technique can be used to detect the location of concrete defects; however, there is no standardized method [30].

A study found that graphene oxide (GO), a nanomaterial, acts as a bridge between hydration products (C-S-H) inside cementitious composites and impacts the compressive strength, bending strength, and tensile strength of cementitious composites according to the mixing concentration [31]. MWCNT networks and MWCNT agglomerates form between the hydration products inside the mortar when mixed with the MWCNT nanomaterial, influencing heat generation and mechanical performance [15]. MWCNT networks form between hydration products and impact the internal pores when mixing MWCNTs in reinforced concrete, thus changing the bond stress between rebar and concrete [32]. Ultrasonic pulse velocity and the waveform of ultrasonic waves, a type of elastic wave, were found to change with the internal state of the specimens, the medium. Shah et al. [33] studied a useful contribution to the ultrasonic non-destructive evaluation of both micro and macro-scale defects or damages induced in concrete under initial and peak-level load applications, respectively. Jost et al. [34] presented a nondestructive inspection method using ultrasonic wave technology to identify phase change regions and infer the state of a material. “Specifications for Structural Concrete (ACI 301)” [35] contains stipulations related to changes in strength according to curing conditions, such as concrete curing days and water-cement ratio.

Researchers have performed numerous studies on estimating the engineering properties (e.g., strength) of concrete and detecting defects using ultrasonic testing. While ultrasonic tests exhibit excellent defect detection performance, analysis of concrete structures mixed with nanomaterials is not sufficient. To bridge this knowledge gap, this study implements defects in nano-cementitious composites and measures the ultrasonic pulse velocity to analyze the defect detection performance. Furthermore, the non-destructive defect detection performance is verified using ultrasonic pulse velocity through a comparison with the compressive strength test results.

## 2. Experimental Procedures

The mechanical performance of the cementitious composite is influenced by the mixed material, internal defect size, and curing days. Therefore, the mixing of nanomaterials, defect size, and curing days were set as the parameters for the ultrasonic non-destructive analysis of cementitious composite mixed with nanomaterials in this study. “Specifications for Structural Concrete (ACI 301)” [35] stipulates a minimum of 28 curing days to develop the design code strength. Accordingly, this study performed curing until 30 days to secure sufficient design code strength and conducted ultrasonic non-destructive tests during the curing period.

Research shows that when defects that may occur during the curing process are implemented by size with arbitrary materials, the non-destructive detection results vary with the defect size [5]. Additionally, the strength decreased as defects and porosity increased when the defect is simplified to the inner center of the specimen to analyze the strength according to defect size in cement-based structures [36]. Based on these research findings, this study implemented the defect in the center of cube-shaped specimens using arbitrary materials and analyzed the mechanical performance and non-destructive detection signals with varying defect sizes.

**Figure 1:**
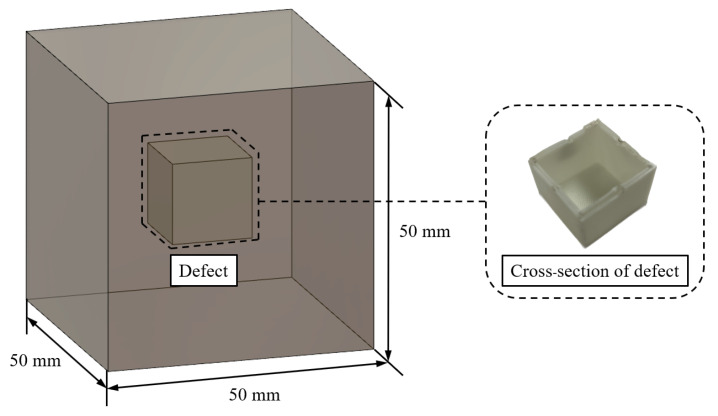
Dimension of nano-cementitious composite for the ultrasonic non-destructive test.

Figure 1 shows the specimens for the ultrasonic non-destructive test. The defects were implemented with polylactic acid (PLA) plastic material. The defect’s outer wall thickness was set to within 2 mm, and the interior used a hollow design to minimize the influence of the plastic material in the non-destructive tests, as shown in the cross-section. In accordance with the “Standard Test Method for Compressive Strength of Hydraulic Cement Mortars (ASTM C 109)” [37], the specimens were fabricated with a size of 50 mm × 50 mm × 50 mm, and the defect was implemented at the inner center of the specimen. Table 1 shows the specimen names and parameters and the volume ratio of defect size to specimen size. The first letter in the specimen name indicates the defect size as follows: D0 (0 × 0 × 0 ${mm}^{3}$), D5 (5 × 5 × 5 ${mm}^{3}$), D10 (10 × 10 × 10 ${mm}^{3}$), D15 (15 × 15 × 15 ${mm}^{3}$), D20 (20 × 20 × 20 ${mm}^{3}$), and D25 (25 × 25 × 25 ${mm}^{3}$). The second letter indicates the MWCNT mixing concentration relative to the weight of cement: C0.0 (0 wt%) and C1.0 (1.0 wt%). Research findings show that when the MWCNT mixing concentration is 1.0 wt% relative to the weight of cement when voltage is supplied, the strength of the specimen is secured while achieving the best electrical conductivity of the nano-cementitious composite [38]. As such, the MWCNT concentration was set to 1.0 wt% in this study.

**Figure 2:**
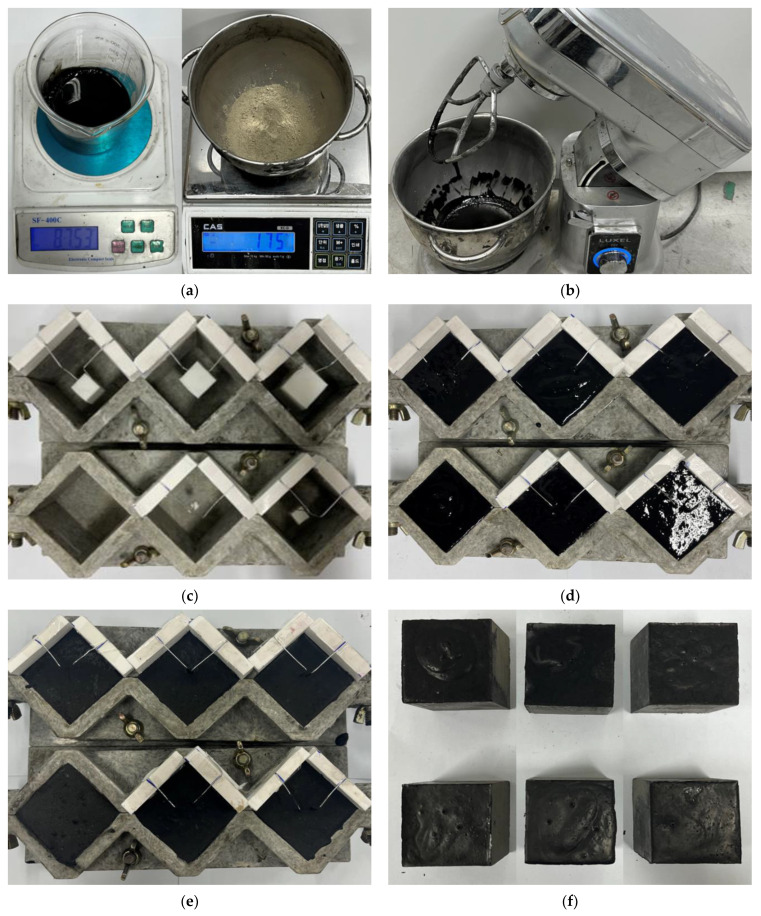
The fabrication process of nano-cementitious composites: (**a**) Measuring the material; (**b**) mixing; (**c**) implementing defects; (**d**) pouring paste in a mold; (**e**) curing on standard conditions; (**f**) specimens with a defect.

Figure 2 shows the fabrication process of the nano-cementitious composite specimens. The water/cement ratio used to fabricate the specimens were set to 1:2 with reference to the “Guide to Curing Concrete (ACI 308)” and measured as shown in Figure 2a [39]. Figure 2b shows the process of mixing the cement and MWCNTs with a mixer for 3 min. Type I ordinary Portland cement and Ditto Technology’s MWCNTs were used for the mixed materials. The defect was implemented in the center of the mold with a size of 50 mm × 50 mm × 50 mm, as shown in Figure 2c. The nano-cementitious paste was poured into the mold after mixing (Figure 2d). Figure 2e shows the curing process of a nano-cementitious composite specimen implemented with an internal defect, and Figure 2f shows a specimen for the ultrasonic non-destructive test. Three specimens were fabricated for each parameter. Human exposure to MWCNTs is considered unlikely while MWCNTs are dispersed in a liquid or embedded in a cementitious composite. However, respirable MWCNTs particles might be released during production, processing, or demolition of cementitious composites. Therefore, MWCNTs should be used with care for respiratory protection [40]. In accordance with the “Standard Practice for Making and Curing Concrete Test Specimens in the Field (ASTM C 31)” [41], curing was performed in a controlled laboratory at a constant temperature (20 ± 5 °C).

**Figure 3:**
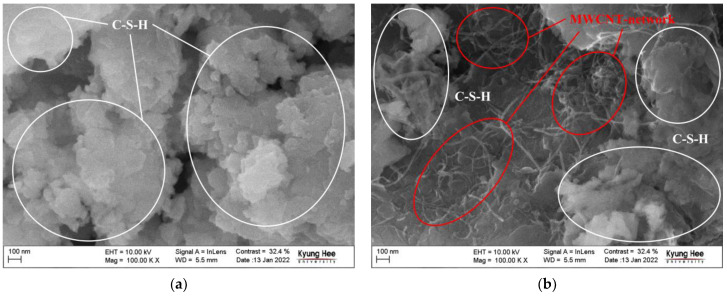
FE-SEM photograph of nano-cementitious composites: (**a**) MWCNT 0.0 wt%; (**b**) MWCNT 1.0 wt%.

The microstructure was confirmed by FE-SEM images to understand the dispersibility of the MWCNTs of the specimen used in the experiments. The photography was performed on LEO SUPRA 55 (Carl Zeiss, Germany). LEO SUPRA 55 is a scanning electron microscope (SEM) with a field emission electron gun. The equipment is able to handle a wide variety of samples, from conducting and semiconducting materials to large, beam-sensitive or non-conducting samples. It has a resolution of 1–4 nm and secondary electron, backscattered, and in-lens imaging modes. It can work at accelerating voltages from 100 V to 30 kV. Figure 3a shows the internal microstructure of the cementitious composite without MWCNTs. Figure 3b shows the internal microstructure of the nano-cementitious composite with MWCNTs. The white circle of the FE-SEM image represents the cement hydration product (C-S-H) formed inside the specimen, and the red circle represents the MWCNTs dispersed inside the specimen. It was confirmed that the nano-cementitious composite had rod-shaped MWCNTs connected between C-S-H. This study confirmed the C-S-H and MWCNT network in the FE-SEM image and analyzed that the MWCNTs were evenly dispersed inside the specimen.

**Figure 4:**
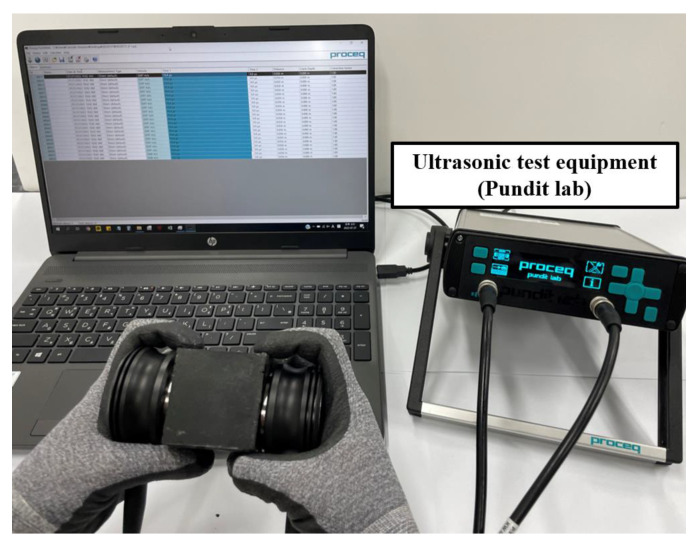
Ultrasonic non-destructive test set-up.

This study conducted ultrasonic non-destructive tests to analyze the defects inside the cementitious composites. Figure 4 shows the ultrasonic non-destructive test process of the specimens. Furthermore, ultrasonic pulse velocity was measured 10 times for each of the three specimens with Pundit Lab (Proceq, Switzerland), an ultrasonic measurement equipment from Proceq. Pundit Lab is a flexible ultrasonic pulse velocity test equipment designed for laboratory operations, and its measuring range is up to 15 m depending on concrete quality. According to the analysis results of the correlation coefficient between ultrasonic pulse velocity and compressive strength according to the type of concrete aggregate, the UPV method was presented as a general method applicable to inspection regardless of the material properties in the concrete structure [42]. Researchers detected micro-defects through concrete ultrasonic analysis when using a 500 kHz transducer [43,44]. Accordingly, this study used a 500 kHz transducer to classify micro-defects and applied a gel ultrasonic couplant to the cross-section of the specimen to increase contact between the specimen and the transducer. The ultrasonic pulse velocity of specimens with internal defects of various sizes was compared to derive the trend according to internal defect size, and the properties were analyzed according to nanomaterial mixing and curing days.

**Figure 5:**
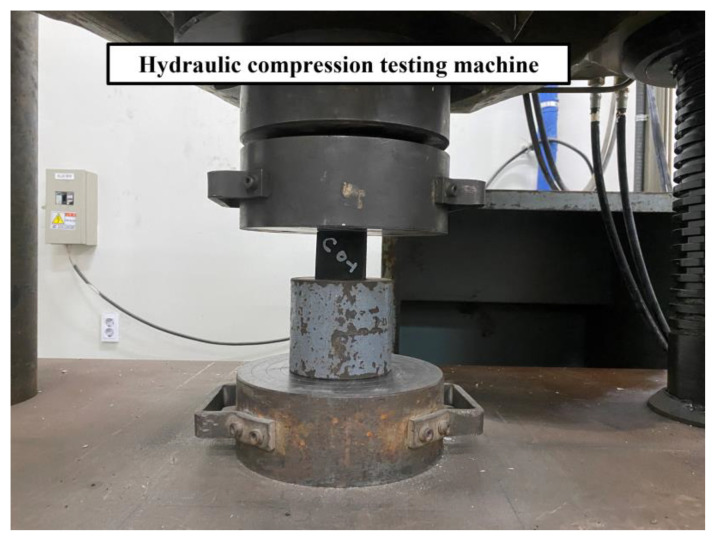
Compressive strength test set-up.

Figure 5 shows the compressive strength test set-up of the specimen. In accordance with the “Standard Test Method for Compressive Strength of Hydraulic Cement Mortars (ASTM C 109)” [37], the compressive strength test was performed with the displacement control (1 mm/min) method using a hydraulic compression testing machine. The experiments were performed on a UH-200A digital servo-hydraulic universal testing machine, with a capacity of 2000 kN. The measured compressive strength varied with the properties of the specimen, and the correlation between ultrasonic pulse velocity and compressive strength was analyzed based on defect size.

## 3. Results and Discussion

### 3.1. Ultrasonic Pulse Velocity

**Figure 6:**
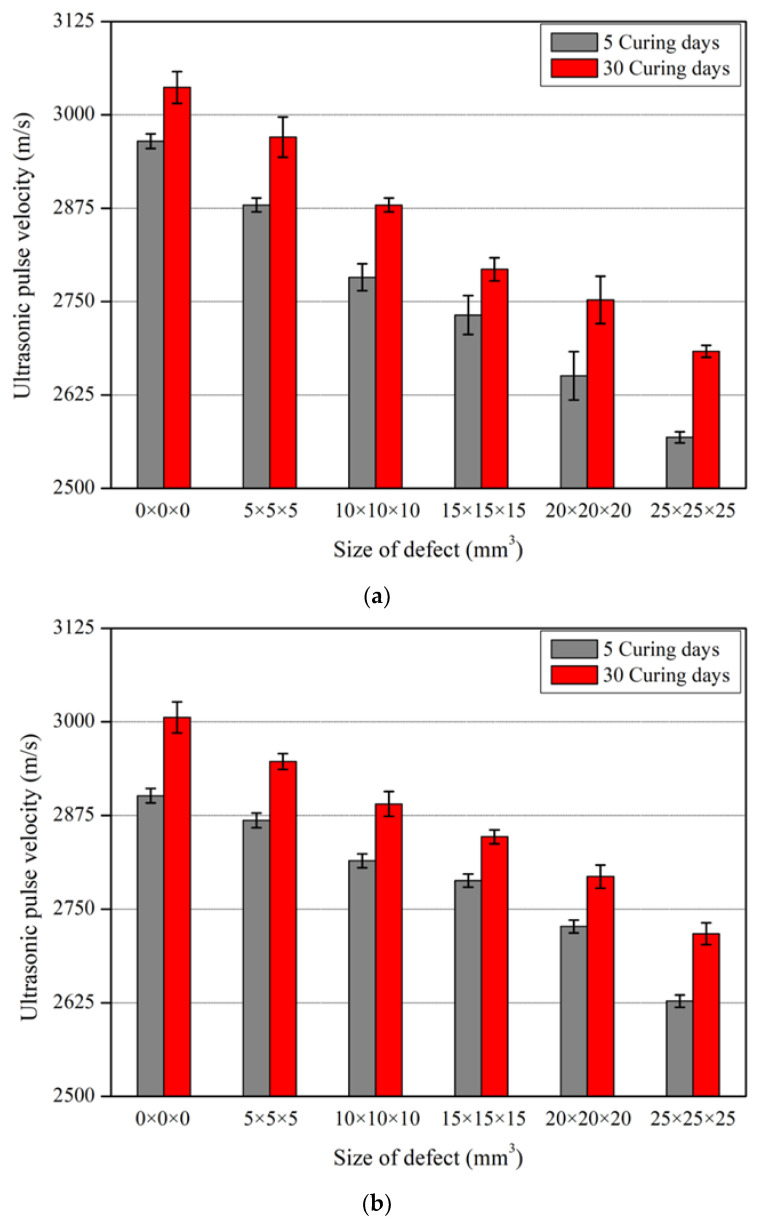
Ultrasonic pulse velocity according to defect size: (**a**) MWCNT 0.0 wt%; (**b**) MWCNT 1.0 wt%.

The ultrasonic pulse velocity of the nano-cementitious composite was measured 10 times for all three specimens and expressed as the mean. The measured values were distributed within ±3% of the mean on all curing days. Figure 6 shows the ultrasonic pulse velocity according to defect size in the nano-cementitious composite at 5 and 30 curing days. Figure 6a shows the test results of the specimens without MWCNTs. The ultrasonic pulse velocity decreased as the defect size increased at 5 and 30 curing days, and at all defect sizes, the ultrasonic pulse velocity at 30 curing days was higher than at 5 curing days. Figure 6b shows the experimental results of the specimens mixed with MWCNTs. Like the above results, the ultrasonic pulse velocity decreased as the defect size increased at 5 and 30 curing days. Furthermore, the ultrasonic pulse velocity was higher at 30 curing days than that at 5 curing days without the influence of defect size. The ultrasonic pulse velocity of the specimens without MWCNTs was higher than that of the specimens with MWCNTs at defect sizes of 0 × 0 × 0 ${mm}^{3}$ and 5 × 5 × 5 ${mm}^{3}$, though the ultrasonic pulse velocity was measured to be lower at other defect sizes. The ultrasonic pulse velocity decreased as the defect size increased when MWCNTs were mixed in the cementitious composite. There is a variation in the ultrasonic pulse velocity when CNT is mixed into a nano-cementitious composite because CNT agglomeration occurs and affects the internal structure. Thus, the nano-cementitious composite is predicted to be less affected by ultrasonic pulse velocity from over a certain defect size.

**Figure 7:**
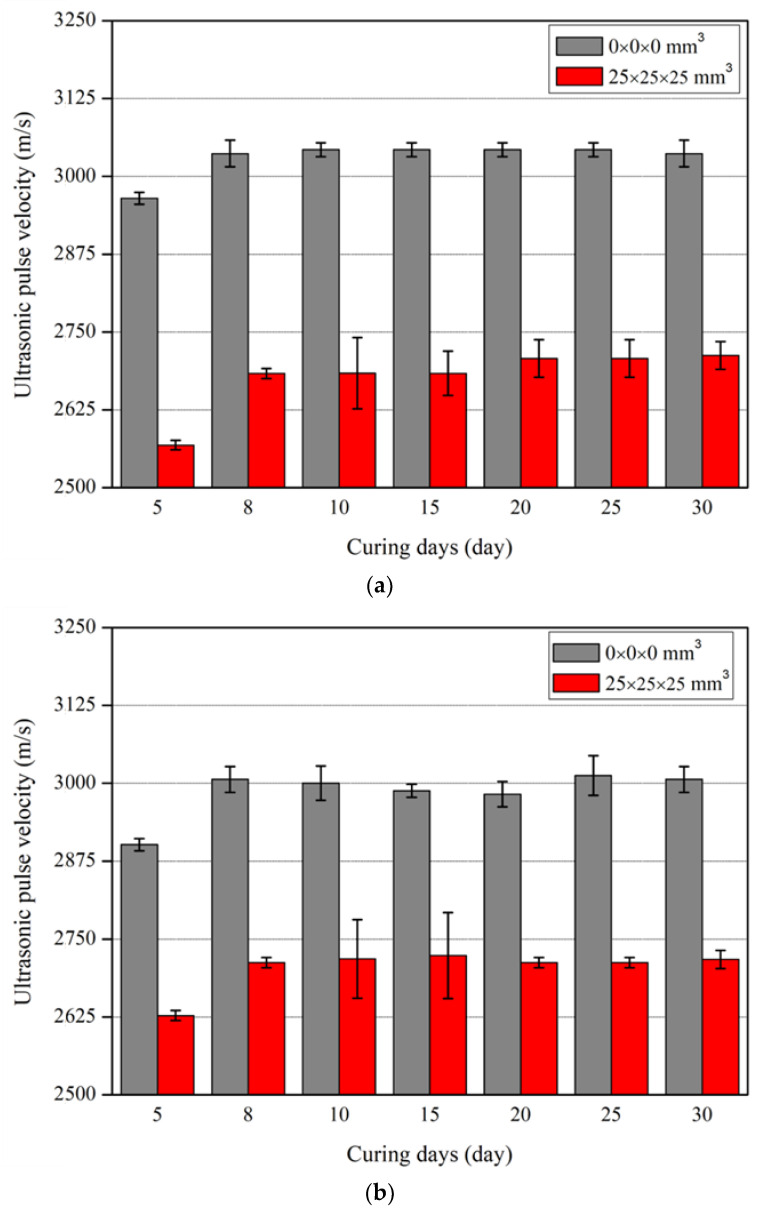
Ultrasonic pulse velocity according to curing days: (**a**) MWCNT 0.0 wt%; (**b**) MWCNT 1.0 wt%.

Figure 7 shows the ultrasonic pulse velocity according to curing days in the specimens without defects and with defects at a size of 25 × 25 × 25 ${mm}^{3}$. Figure 7a shows the experimental results of the specimens without MWCNTs. At 8 curing days, the ultrasonic pulse velocity increased up to 4.5% in relation to that at 5 curing days. After 8 curing days, the ultrasonic pulse velocity maintained a constant value without the influence of defect size. Figure 7b shows the experimental results of the specimens with MWCNTs. At 8 curing days, the ultrasonic pulse velocity increased up to 3.6% in relation to that at 5 curing days. The ultrasonic pulse velocity of the nano-cementitious composite mixed with MWCNTs maintained a constant value after 8 curing days, both in the case of no defect and a defect of 25 × 25 × 25 ${mm}^{3}$. Hence, the ultrasonic pulse velocity was confirmed to maintain a constant value after 8 curing days in all specimens, which is attributed to the internal hardening of the cementitious composite according to the hydration process.

**Figure 8:**
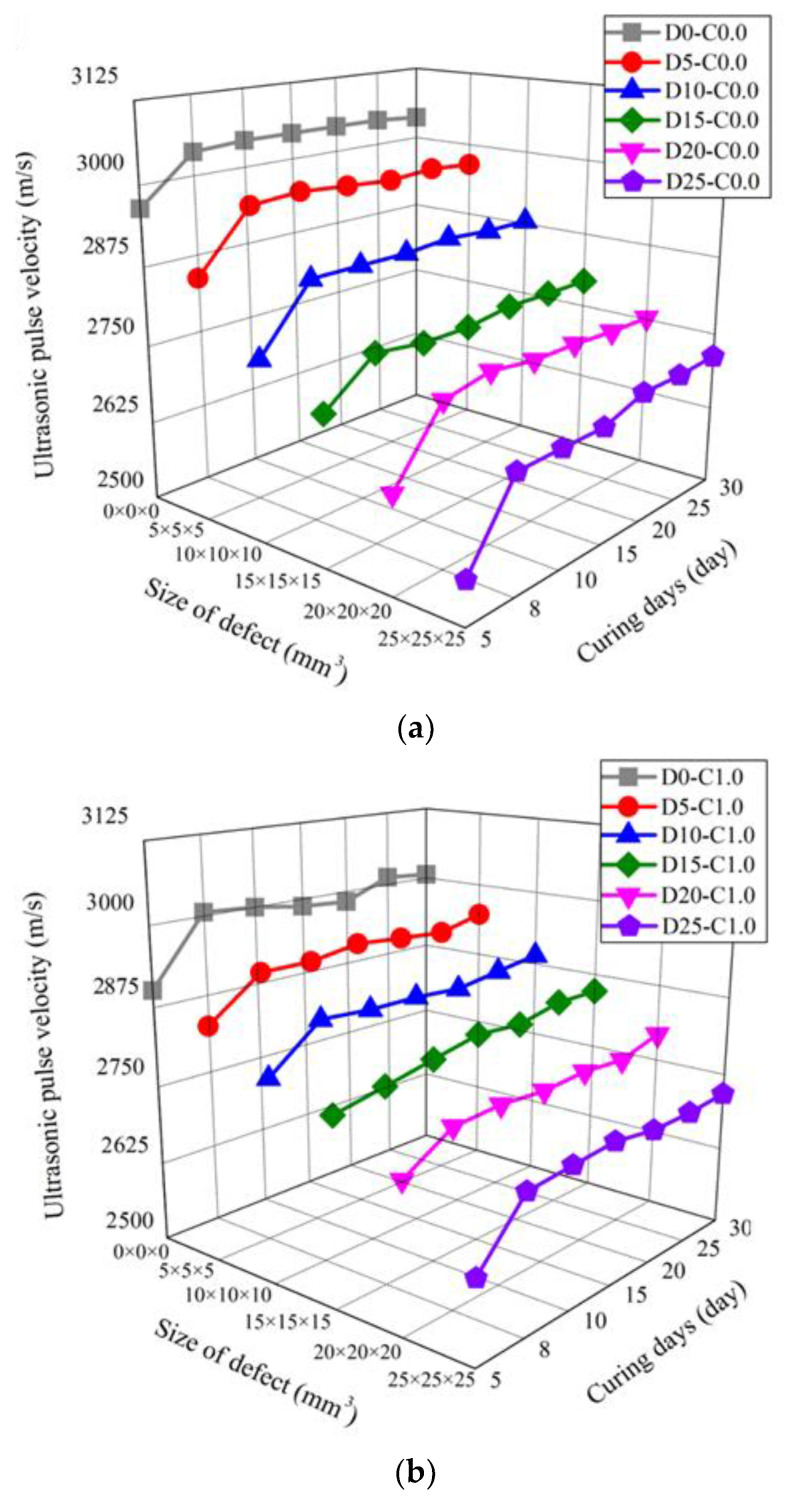
Ultrasonic pulse velocity according to defect size and curing days: (**a**) MWCNT 0.0 wt%; (**b**) MWCNT 1.0 wt%.

Figure 8 presents the ultrasonic test results according to all curing days and six defect sizes for the nano-cementitious composites. Table 2 shows the ultrasonic pulse velocity of the nano-cementitious composites at 30 curing days. Figure 8a shows the experimental results of the cementitious composite without MWCNTs. At 30 curing days, the ultrasonic pulse velocity was 3037 m/s in D0_C0.0, 2965 m/s in D5_C0.0, and 2879 m/s in D10_C0.0. Additionally, it was measured at 2793 m/s in D15_C0.0, 2752 m/s in D20_C0.0, and 2712 m/s in D25_C0.0. Figure 8b shows the experimental results of the nano-cementitious composite mixed with MWCNTs. At 30 curing days, the ultrasonic pulse velocity was 3006 m/s in D0_C1.0, 2947 m/s in D5_C1.0, and 2890 m/s in D10_C1.0. Additionally, it was measured at 2846 m/s in D15_C1.0, 2793 m/s in D20_C1.0, and 2717 m/s in D25_C1.0. The ultrasonic pulse velocity decreased as defect size increased at all measured curing days regardless of whether MWCNTs were mixed. When the defect size was 12.5% of the specimen volume, the ultrasonic pulse velocity decreased by up to 10.7% in the specimen without MWCNTs and up to 9.6% in the specimen mixed with MWCNTs. Regardless of whether MWCNTs were mixed in the nano-cementitious composite at 8 curing days, the ultrasonic pulse velocity maintained a constant level at all defect sizes. These findings indicate that the influence of the MWCNT nanomaterial on ultrasonic pulse velocity is negligible and that there is no problem with performing ultrasonic non-destructive tests to detect defects in the specimens mixed with MWCNTs.

### 3.2. Compressive Strength

**Figure 9:**
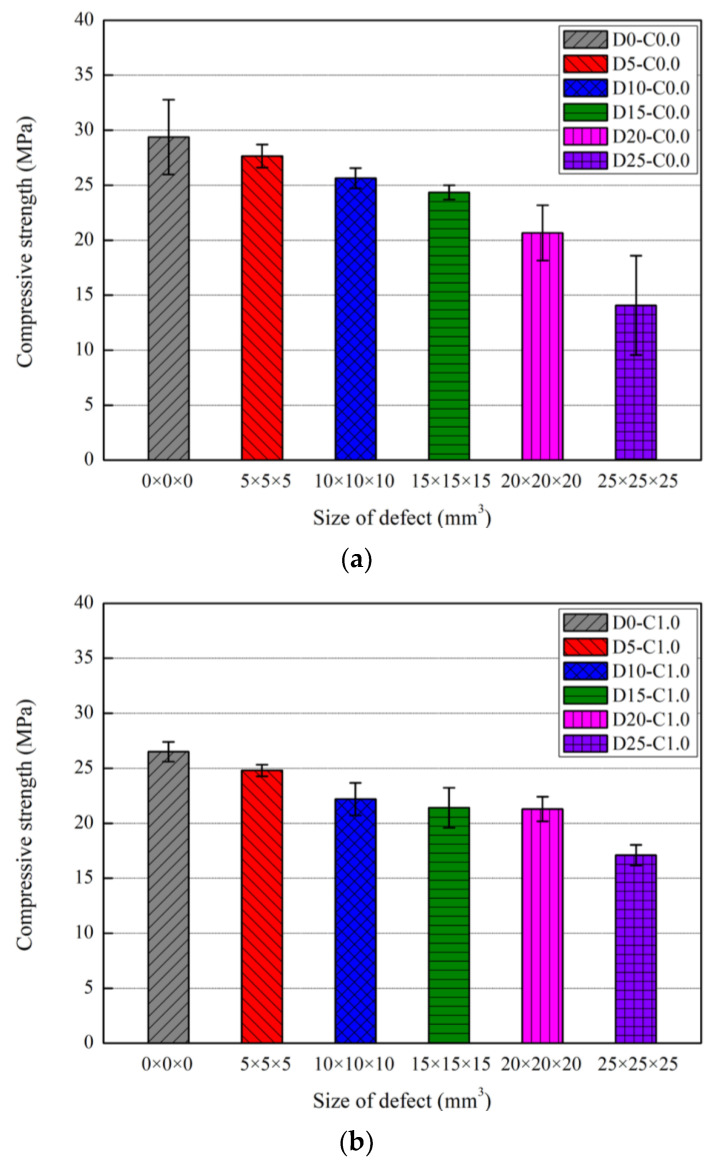
Compressive strength according to defect size: (**a**) MWCNT 0.0 wt%; (**b**) MWCNT 1.0 wt%.

Figure 9 and Table 3 show the compressive strength test results according to defect size at 30 curing days of the nano-cementitious composite. The mean compressive strength of three specimens was expressed with an error bar. In the specimens without MWCNTs, the compressive strength was measured at 29.4 MPa without a defect, 27.7 MPa with a 5 × 5 × 5 ${mm}^{3}$ defect, 25.6 MPa with a 10 × 10 × 10 ${mm}^{3}$ defect, 24.4 MPa with a 15 × 15 × 15 ${mm}^{3}$ defect, 20.7 MPa with a 20 × 20 × 20 ${mm}^{3}$ defect, and 14.1 MPa with a 25 × 25 × 25 ${mm}^{3}$ defect. In the nano-cementitious composites with MWCNTs, the compressive strength was measured at 26.6 MPa without a defect, 24.8 MPa with a 5 × 5 × 5 ${mm}^{3}$ defect, 22.2 MPa with a 10 × 10 × 10 ${mm}^{3}$ defect, 21.4 MPa with a 15 × 15 × 15 ${\mathrm{mm}}^{3}$ defect, 21.3 MPa with a 20 × 20 × 20 ${mm}^{3}$ defect, and 17.1 MPa with a 25 × 25 × 25 ${mm}^{3}$ defect. Thus, comparing the cases without a defect, the measured compressive strength was lower in the specimen mixed with MWCNTs. The compressive strength and mixing concentration were proportional up to 0.5 wt% of cement weight when MWCNTs are mixed with the cementitious composite. However, after 0.5 wt% of cement weight, the compressive strength declines due to agglomeration caused by van der Waals force between the MWCNT particles [32]. The best heat generation and electrical performance were yielded when mixing MWCNTs in the cementitious composite at 1.0 wt% of cement weight [15]. Focusing on this result, MWCNTs were mixed at 1.0 wt% of cement weight, and the compressive strength was found to be somewhat low due to agglomeration. The compressive strength of the nano-cementitious composite decreased as the defect size increased regardless of whether MWCNTs were mixed. When the defect size was 12.5% of the specimen volume, the compressive strength decreased by up to 52.1% in the specimen without MWCNTs and up to 35.7% in the specimen mixed with MWCNTs.

**Figure 10:**
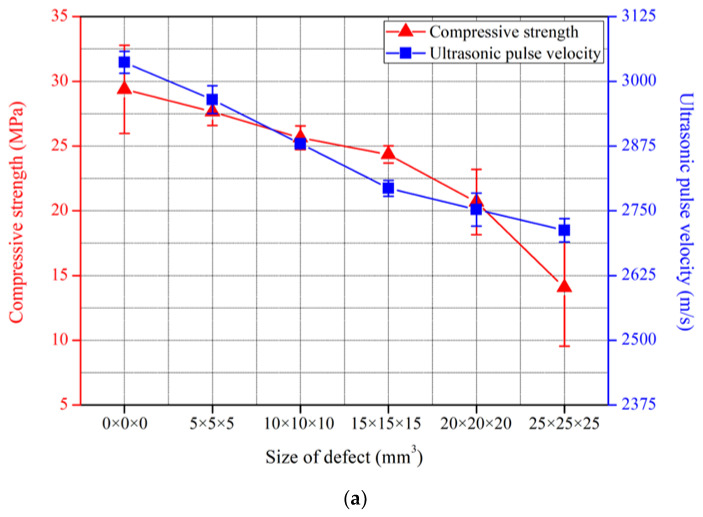

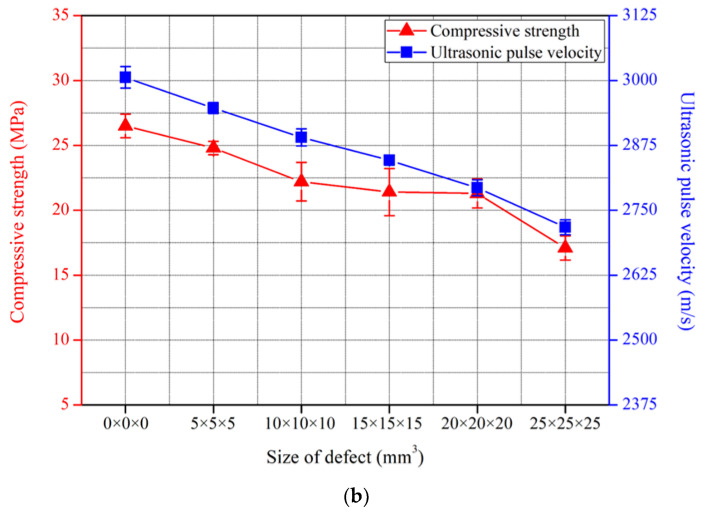
Comparison between compressive strength and ultrasonic pulse velocity: (**a**) MWCNT 0.0 wt%; (**b**) MWCNT 1.0 wt%.

In this study, the compressive strength of all specimens decreased as the defect size increased, indicating that defects can be identified based on the compressive strength. Figure 10 illustrates the correlation between compressive strength and ultrasonic pulse velocity according to defect size in the nano-cementitious composite at 30 curing days. Figure 10a shows the compressive strength and ultrasonic pulse velocity of the cementitious composite without MWCNTs in an error bar, and Figure 10b shows that of the nano-cementitious composite mixed with MWCNTs. In the nano-cementitious composite, both compressive strength and ultrasonic pulse velocity tended to decrease as the defect size increased. Compared to previous studies, these results have a similar tendency to the strength variation with defect sizes, and the tendency can be seen in ultrasonic pulse velocity. This study identified the correlation between ultrasonic pulse velocity and compressive strength according to the defect size in all cases. Thus, the ultrasonic non-destructive test can infer defects produced inside nano-cementitious composites according to the size, and compressive strength can be deduced based on the correlation.

## 4. Conclusions

This study conducted ultrasonic non-destructive tests to analyze cementitious composites according to nanomaterial mixing, defect size, and curing days and identified the correlations with compressive strength. The following conclusions were drawn.

1. The ultrasonic pulse velocity of the general cementitious composite without MWCNTs was found to change according to the curing days and defect size. The ultrasonic pulse velocity maintained a constant value and decreased with increasing defect size at all curing days in all cases after 8 curing days even though the ultrasonic pulse velocity increased at the beginning of curing. The ultrasonic pulse velocity declined by up to 10.7% when the defect size was 12.5% of the specimen volume. When defects occur inside, the cementitious composite’s properties, which is a medium of ultrasonic, vary. Therefore, the ultrasonic pulse velocity also changes. This indicates that the defect size can be deduced by comparing specimens with and without defects;
2. The nano-cementitious composite mixed with MWCNTs showed a similar trend to the general cementitious composite, and the ultrasonic pulse velocity changed according to the curing days and defect size. The ultrasonic pulse velocity increased at the beginning of curing and then maintained a constant value after 8 days of curing. In the nano-cementitious composite, the ultrasonic pulse velocity declined by up to 9.6% when the defect size was 12.5% of the specimen volume. According to the previous results, it is determined that even if MWCNTs are mixed into the cementitious composites, the effect on the ultrasonic pulse velocity is negligible. This suggests that defects can be detected even when applying the existing ultrasonic non-destructive test method to nano-cementitious composites;
3. The compressive strength decreased with increasing defect size in all cases. When the defect size was 12.5% of the specimen volume, it decreased by up to 52.1% in the general cementitious composite and up to 35.7% in the nano-cementitious composite. It was found that, as the defect size increased, the cross-sectional area of the specimen decreased, resulting in a decrease in compressive strength. In this study, the compressive strength of all specimens decreased as the defect size increased, indicating that defects can be identified according to size based on compressive strength;
4. The ultrasonic pulse velocity and compressive strength were distinguished according to the internal defect size even when nanomaterials were mixed in the cementitious composite. The ultrasonic pulse velocity and compressive strength showed a similar tendency to decrease as the defect size increased, and a correlation according to the defect size was confirmed. Therefore, it will be possible to predict defects in nano-cementitious composites and infer mechanical performance when the result data of ultrasonic non-destructive tests and compressive strength tests are accumulated in the future, based on this correlation.

## Figures and Tables

**Table 1:** Specimen names and experimental parameters.

Specimen Name	Size of Defects	Volume Ratio of Defect Size to Specimen Size	MWCNT Concentration
D0_C0.0	0 × 0 × 0 mm^3^	0.0%	0.0 wt%
D5_C0.0	5 × 5 × 5 mm^3^	0.1%	
D10_C0.0	10 × 10 × 10 mm^3^	0.8%	
D15_C0.0	15 × 15 × 15 mm^3^	2.7%	
D20_C0.0	20 × 20 × 20 mm^3^	6.4%	
D25_C0.0	25 × 25 × 25 mm^3^	12.5%	
D0_C1.0	0 × 0 × 0 mm^3^	0.0%	1.0 wt%
D5_C1.0	5 × 5 × 5 mm^3^	0.1%	
D10_C1.0	10 × 10 × 10 mm^3^	0.8%	
D15_C1.0	15 × 15 × 15 mm^3^	2.7%	
D20_C1.0	20 × 20 × 20 mm^3^	6.4%	
D25_C1.0	25 × 25 × 25 mm^3^	12.5%	

**Table 2:** Ultrasonic pulse velocity of nano-cementitious composite on 30 curing days.

Specimen Name	Ultrasonic Pulse Velocity	Reduction Ratio Compare to Non-Defected Specimen
D0_C0.0	3037 m/s	-
D5_C0.0	2965 m/s	−2.4%
D10_C0.0	2879 m/s	−5.2%
D15_C0.0	2793 m/s	−8.0%
D20_C0.0	2752 m/s	−9.4%
D25_C0.0	2712 m/s	−10.7%
D0_C1.0	3006 m/s	-
D5_C1.0	2947 m/s	−2.0%
D10_C1.0	2890 m/s	−3.9%
D15_C1.0	2846 m/s	−5.3%
D20_C1.0	2793 m/s	−7.1%
D25_C1.0	2717 m/s	−9.6%

**Table 3:** Compressive strength of nano-cementitious composite on 30 curing days.

Specimen Name	Compressive Strength	Reduction Ratio Compare to Non-Defected Specimen
D0_C0.0	29.4 MPa	-
D5_C0.0	27.7 MPa	−5.9%
D10_C0.0	25.6 MPa	−12.7%
D15_C0.0	24.4 MPa	−17.1%
D20_C0.0	20.7 MPa	−29.7%
D25_C0.0	14.1 MPa	−52.1%
D0_C1.0	26.6 MPa	-
D5_C1.0	24.8 MPa	−6.6%
D10_C1.0	22.2 MPa	−16.4%
D15_C1.0	21.4 MPa	−19.3%
D20_C1.0	21.3 MPa	−19.9%
D25_C1.0	17.1 MPa	−35.7%

## Data Availability

No new data were created or analyzed in this study. Data sharing is not applicable to this article.
